# The interplay between genetic variation and gene expression of the glucocorticoid receptor gene NR3C1 and blood cortisol levels on verbal memory and hippocampal volumes

**DOI:** 10.1007/s00406-022-01420-w

**Published:** 2022-05-17

**Authors:** Sandra Van der Auwera, Johanna Klinger-König, Katharina Wittfeld, Jan Terock, Anke Hannemann, Robin Bülow, Matthias Nauck, Uwe Völker, Henry Völzke, Hans Jörgen Grabe

**Affiliations:** 1 Department of Psychiatry and Psychotherapy, University Medicine Greifswald, Ellernholzstraße 1-2, 17489 Greifswald, Germany; 2 German Center for Neurodegenerative Diseases (DZNE), Site Rostock/Greifswald, Greifswald, Germany; 3 Department of Psychiatry and Psychotherapy, HELIOS Hanseklinikum Stralsund, Rostocker Chaussee, Stralsund, Germany; 4 Institute of Clinical Chemistry and Laboratory Medicine, University Medicine Greifswald, Greifswald, Germany; 5 German Centre for Cardiovascular Research (DZHK), Partner Site Greifswald, University Medicine Greifswald, Greifswald, Germany; 6 Department of Diagnostic Radiology and Neuroradiology, University Medicine Greifswald, Greifswald, Germany; 7 Interfaculty Institute for Genetics and Functional Genomics, University Medicine Greifswald, Greifswald, Germany; 8 Institute for Community Medicine, University Medicine Greifswald, Greifswald, Germany

**Keywords:** NR3C1, Verbal memory, Hippocampus, rs56149945, Cortisol, Glucocorticoid receptor

## Abstract

The hypothalamus–pituitary–adrenal axis is the main physiological stress response system and regulating the release of cortisol. The two corticoid receptors encoded by the genes *NR3C1* and *NR3C2* are the main players in regulating the physiological response to cortisol. This biological system has been linked to neurocognitive processes and memory, yet the mechanisms remain largely unclear. In two independent general population studies (SHIP, total sample size > 5500), we aim to diseantangle the effects of genetic variation, gene expression and cortisol on verbal memory and memory associated brain volume. Especially for *NR3C1* results exhibited a consistent pattern of direct an interactive effects. All three biological layers, genetic variation (rs56149945), gene expression for *NR3C1* and cortisol levels, were directly associated with verbal memory. Interactions between these components showed significant effects on verbal memory as well as hippocampal volume. For *NR3C2* such a complex association pattern could not be observed. Our analyses revealed that different components of the stress response system are acting together on different aspects of cognition. Complex phenotypes, such as cognition and memory function are regulated by a complex interplay between different genetic and epigenetic features. We promote the glucocorticoid receptor *NR3C1* as a main target to focus in the context of verbal memory and provided a mechanistic concept of the interaction between various biological layers spanning *NR3C1* function and its effects on memory. Especially the *NR3C1* transcript seemed to be a key element in this complex system.

**Supplementary Information:**

The online version contains supplementary material available at 10.1007/s00406-022-01420-w.

## Introduction

Acute stress impacts negatively on cognition and memory [1]. One of the main physiological stress-response systems is the hypothalamus–pituitary–adrenal (HPA) axis. Inter alia, the HPA axis is initiating the release of the glucocorticoid cortisol by the adrenal gland. The mineralocorticoidreceptor (MR) and the glucocorticoidreceptor (GR) are two major players of the HPA axis which especially become activated by cortisol. The release of cortisol has a strong circadian rhythm and is increasing in response to psychological stress. Both receptors strongly differ in their affinity to cortisol with the MR showing a ten-fold increased affinity to cortisol compared to the GR. The GR becomes mainly occupied when cortisol levels are high, e.g., during circadian peak or after stress. In the latter case, it acts as a key mechanism to fastly activate a physiological stress response [2]. In contrast, the MR is largely occupied under basal conditions and by that regulating gene expression also in the absence of stress. Both receptors are important regulators of the HPA axis and for the physiological adaption to stress while acting as transcription factors to alter gene expression of multiple neurochemical target systems [3]. Alterations of the HPA axis are involved in the pathophysiology of many psychiatris stress-related disorders, such as depression, anxiety disorders, post-traumatic stress disorder or schizophrenia [4, 5].

The receptors are expressed by the genes *NR3C1/GR* (nuclear receptor subfamily 3 group C member 1) and *NR3C2/MR* (nuclear receptor subfamily 3 group C member 2) which are found almost ubiquitously in human tissues but are highly expressed in the brain, especially in the hypothalamus and hippocampus [3, 6].

Both genes have been associated with memory performance in the past but mostly in the context of emotional memory, stress or comorbid psychiatric disorders like depression or post-traumatic stress disorder [7–9]. El-Hage and colleagues [10] found that genetic variants in *NR3C1* had an effect on the dorsolateral prefrontal cortex involved in working memory function. The BclI polymorphism of *NR3C1* was found to be associated with emotional memory performance, but not neutral memory performance, in healthy individuals [11]. For *NR3C2* there is also evidence for an association with memory function. The A-allele of the single nucleotide polymorphism (SNP) rs5534 was associated with negative memory bias in subjects who experienced childhood trauma [12, 13]. Additionally, our group could recently demonstrate the effect of two *NR3C2* SNPs (rs5522 and rs2070951) on memory decline in a longitudinal general population sample [14]. The SNP rs2070951 was also found to be involved in multiple memory systems by influencing cortisol secretion [15].

On the level of gene expression, both genes could also be linked to cognition and memory. Vukojevic and collegues [8] found an association between increased expression of the *NR3C1* gene and traumatic memory and post-traumatic stress disorder. Likewise, in mice overexpression of *NR3C2* affected spatial memory performance and enhanced contextual fear memory formation [16]. Another group found a *NR3C2* haplotype associated with enhanced *NR3C2* expression leading to a stress-induced shift in learning performance [17].

As both genes are targeted by cortisol in response to stress, an involvement of cortisol on memory function seems likely. In a double-blind study [18], cortisol was found to be involved in reconsolidation processes with suppressing cortisol leading to a strengthening of episodic memory. A review [19] confirmed that induced stress was associated with increases in cortisol levels and memory impairment. On the level of distinct brain regions, the hippocampus is the main target region for *NR3C1* and *NR3C2* gene expression in the brain as well as mainly involved in learning and memory function [20, 21]. Thus, the important role of this target region in the stress-response memory cascade needs to be investigated [3].

The detailed mechanisms of how all these factors interact together and thus influence memory function in healthy individuals, is still not sufficiently investigated. Past research mainly focussed on this system in response to external stressors or in subjects with comorbid disorders.

In this study, we investigate the mechanisms of how the main players (genetic features, gene expression and cortisol levels) act on memory and hippocampal volume to diseantangle this complex network.

1. We now aim to explore the association between the SNP rs56149945 of *NR3C1* (also known as p.N363S or rs6195) on verbal memory in two independent samples from the general population. This genetic variant has been associated to HPA axis glucocorticoid sensitivity [22] as well as various stress induced psychiatric disorders [23–25]. Additionally, we intend to explore the role of the glucocorticoid cortisol on this path.
2. In a subcohort with a variety of additional multi-layer biological data available (hippocampal volume, cortisol levels, SNP information, mRNA transcripts) we investigate the direct associations and interactive effects between different biological systems for *NR3C1* (genetics, mRNA transcript and cortisol) on verbal memory and hippocampal volume.
3. In case of significant results for the *NR3C1* SNP and the corresponding mRNA transcripts, we will additionally analyse the *NR3C2* SNP rs2070951 and its corresponding mRNA transcript to detect similarities and differences of the two systems. We selected rs2070951 as marker SNP for *NR3C2* as we had previously identified this SNP to be involved in verbal memory decline [14].

## Materials and methods

### Sample

We analyzed data from the Study of Health in Pomerania (SHIP) [26] comprising adult German residents in northeastern Germany. A number of 4308 Caucasian subjects participated at baseline (SHIP-START-0; 1997–2001). To date, three regular follow‐ups have been carried out (SHIP-START‐1/2/3). In parallel to SHIP-START-2 (hereafter referred to as START-2), detailed assessments of life events and mental disorders were conducted within the SHIP‐LEGENDE (hereafter referred to as LEGENDE) study (Life Events and Gene–Environment Interaction in Depression) from 2007 to 2010 including *n* = 2400 participants from the SHIP-START-0 sample [27]. From 2008 to 2012, a new independent sample called SHIP-TREND-0 (*n* = 4420; hereafter referred to as TREND) from the same area was drawn, encompassing similar examinations like SHIP-START [26]. Education measured as the number of schooling years was divided into < 10/ = 10/ > 10 years (according to the german school system). Smoking status was devided into never, former and current smokers. BMI was measured as (weight in kg)/(height in m)^2^. Hypertension, hypertensive medication and type-2 diabetes were assessed via self-report.

For a graphical overview on the different studies and available variables, see Fig. S1. The investigations in SHIP were carried out in accordance with the Declaration of Helsinki, including written informed consent of all participants. The survey and study methods were approved by the institutional review board of the University of Greifswald.

### Instruments

#### Verbal memory

Amongst others, subjects in LEGENDE were administered the auditory Verbal Learning and Memory Test (VLMT), a German adaption of the widely used Rey Auditory Verbal Learning Test [28]. The VLMT was used to assess short-term learning as well as delayed retrieval [29]. The number of correctly remembered words for short and long-term retrieval was stored in two separate sum scores.

In TREND, the word list of the Nuremberg Age Inventory (NAI) was used as a measure for immediate- and delayed memory performance. The NAI is a German test developed to measure the cognitive abilities during brain aging [29, 30]. The number of correctly identified words is summarized to a sum score minus the number of wrongly identified distractor words for short- and long-term retrieval, respectively.

#### Psychiatric measures

Current depressive symptoms were assessed in LEGENDE using the Beck Depression Inventory (BDI-II), which is a 21-item self-report questionnaire with high reliability and validity [31]. In TREND, the Patient Health Questionnaire (PHQ-9) was used, a 9-item self-report questionnaire also with high reliability and validity [32]. Both sum scores reflect the current level of depressive symptomatic. In LEGEND and TREND, a diagnostic interview for mental disorders was performed based on the Diagnostic and Statistical Manual for Mental Disorders (IV edition) diagnostic criteria which also includes the diagnosis of major depressive disorder (MDD).

Childhood trauma was assessed using the 25-item version of the CTQ, a widely used self-report scale [33]. It comprises five different subscales: emotional abuse, physical abuse, sexual abuse, emotional neglect, and physical neglect. Responses are made on a 5-point Likert-type scale to express the frequency of occurrence and ranges from ‘never true’ to ‘very often true’. The CTQ sumscore was used for analysis.

### Whole blood measurements

For detail on blood measurements including cortisol, see supplement or [34]. As cortisol secretion is influenced by the circadian rhythm, participants were excluded from the analyses if the time of blood sampling was before 7:00 am or after 1:00 pm (TREND: *n* = 1, SHIP-2: *n* = 3). Note, that participants of TREND but not START-2 were asked to fast before blood sampling. Hence, fasting time was longer in TREND (mean (M) = 08:50 h, standard deviation (SD) = 5:20 h) than in START-2 (M = 3:30 h, SD = 2:41 h). Also pregnant woman and subjects taking hormones or synthetic glucocorticoids (ATC G03 and H02) were excluded from the cortisol analyses.

### Genetic data

SNP information was taken from the genetic data in SHIP (described elsewhere [26, 29]).

Imputation of genotypes was performed using the HRCv1.1 reference panel and the Eagle and minimac3 software implemented in the Michigan Imputation Server for pre-phasing and imputation, respectively.

### Whole-blood gene expression data in TREND subsample

Sample collection and whole-blood RNA preparation were described in detail elsewhere and in the supplement [35]. For this study, we extracted exclusively the transcript level data of the two genes *NR3C1* (three transcripts available) and *NR3C2* (only one transcript available). For *NR3C1* and *NR3C2* we selected the transcript whose corresponding DNA sequence contained the SNPs, respectively, resulting in the transcripts NM_000176 (NR3C1 ILMN_2389347) and NM_000901 (NR3C2 ILMN_2210934) (compare Fig. S2/S3). We used the quantile normalised and log2 transformed transcript level quantification for the regression analyses. The mRNA data was available for n = 991 subjects.

### Magnetic resonance imaging data in TREND subsample

Subjects from TREND were asked to participate in a whole-body magnetic resonance imaging (MRI) assessment [36]. After exclusion of medical conditions (e.g., history of cerebral tumor, stroke, Parkinson's Disease, epilepsy) or technical reasons (e.g., severe movement artefacts), data sets were available for 829 subjects. For technical details of the structural magnetic resonance imaging (MRI) data of the head, see supplement. For the present analyses, hippocampal volume in the left and right hemispheres was averaged. Quality control was conducted in accordance to the ENIGMA protocol (http://enigma.ini.usc.edu/protocols/imaging-protocols/).

### Statistical analyses

Subject characteristics were assessed by mean, standard deviation and range for metric variables and with numbers and percentage for categorical data. Sample comparisons were performed using *t* test for metric variables and Chi^2^-test for categorical variables.

1. Ordinary least square regression models with word recall score or cortisol levels as outcome were used assessing the effect of rs56149945 in both cohorts. Genetic analyses were controlled for the effect of age, sex, educational level, current depressive symptoms, three genetic principal components and genetic batch where necessary. As the minor allele frequency of the SNP rs56149945 was < 5% for both samples, we used a dichotomous variable (rs56149945 AA vs. GA/GG). Cortisol level analyses were corrected for time of blood sampling, fasting time, smoking status, alcohol consumption, HbA1c, blood cells (white, red, platelets), waist-to-height ratio, age and sex. As cortisol was available in START-2 and TREND, we calculated these models in both cohorts separately as well as in the combined sample.
2. Analyses assessing direct and interaction effects between SNP rs56149945, mRNA transcript of *NR3C1* and cortisol level on hippocampal volume and verbal memory were performed in a subsample of TREND where all data were available. Gene expression analyses were controlled for technical covariates (time of blood sampling, RNA integrity number as a measure of RNA quality, RNA amplification batch), blood cells (white, red, platelets, mean platelet volume, monocytes, lymphocytes, basophils, neutrophils, eosinophils), haematocrit, smoking status, BMI, educational level, current depressive symptoms, age, and sex. Analyses for brain volume were additionally corrected for intracranial volume (ICV).
3. As an extension of our previous paper on the effect of rs2070951 on verbal memory, we tried to replicate these direct associations and interactions (see above) also for *NR3C2* (SNP and mRNA) using the same statistical models.

Age, time of blood sampling and fasting time were taken non-linear as restricted cubic splines (with four knots) into all models. All p values were assessed via bootstrap with 1000 replicates. A *p* value of *p* < 0.05 was set as significance level. All analyses were calculated on the set of participants with full data for all variables in the specific model which explain varying sample sizes between analyses (see Figs. S1, S4, S5 for details).

## Results

### Sample characteristics

Basic participant characteristics and comparisons of both samples (and the TREND subsample) are given in Table 1. In brief, the two main samples TREND and LEGENDE showed significant differences in age and educational level as well as cortisol. The age differences were caused by the different survey waves of LEGENDE (follow-up of SHIP-START) and TREND (new baseline cohort), the differences in education were caused by the different time periods of the studies (SHIP-START 1997–2001, SHIP-TREND 2008–2012). Many physical measurement variables were not available for LEGENDE as this was a psychiatric study. The TREND subsample (none-diabetic subjects) showed significant differences compared to the full sample in most of the parameters. The subsample was significantly younger, contained more females, was higher educated, had a higher immediate recall score as well as higher cortisol levels.

**Table 1:** Sample characteristics and comparisons between TREND and LEGENDE as well as between the full TREND sample and subsample

Variable	TREND (*n* = 3460**)	LEGENDE (*n* = 2064**)	TREND (subsample *n* = 955**)	Comparison TREND vs. LEGENDE	Comparison TREND full vs. subsample
Age	51.5 (15.3), 20–82	55.5 (13.8), 29–89	50.1 (13.8), 20–81	*T* = − 9.9, *p* < 0.001	*T* = 2.5, *p* = 0.01
Sex				Chi^2^ = 0.54, *p* = 0.46	Chi^2^ = 5.2, *p* = 0.02
Males	1689 (48.5%)	1023 (47.5%)	415 (44.3%)		
Females	1795 (51.5%)	1132 (52.5%)	522 (55.7%)		
Educational level				Chi^2^ = 48.4, *p* < 0.001	Chi^2^ = 43.1, *p* < 0.001
< 10 years	737 (21.2%)	591 (27.4%)	111 (11.9%)		
10 years	1,827 (52.4%)	1,142 (53%)	529 (56.5%)		
> 10 years	920 (26.4%)	422 (19.6%)	297 (31.7%)		
Current depressive symptoms	12.9 (3.6), 9–35	6.5 (7.3), 0–58	12.8 (3.6), 9–35	–	T = 0.6, p = 0.54
MDD lifetime	630 (18.5%)	341 (16.6%)	162 (17.0%)	Chi^2^ = 3.2, p = 0.08	Chi^2^ = 0.95, p = 0.33
CTQ sum score	33.4 (9.7), 25–115	33.8 (9.4), 25–119	32.9 (9.5), 25–111	T = 1.4, p = 0.15	T = 1.3, p = 0.21
Verbal memory				–	
Immediate recall	5.2 (1.3), 0–8	24.5 (6.3), 2–45	5.4 (1.3), 0–8		*T* = − 2.4, *p* = 0.01
Delayed recall	5.7 (1.7), − 3 to 8	7.9 (3.1), 0–15	5.8 (1.7), − 3 to 8		*T* = − 0.9, *p* = 0.34
NR3C1 SNP rs56149945				Chi^2^ = 2.3, *p* = 0.31	Chi^2^ = 0.07, *p* = 0.97
AA	3155 (90.6%)	1973 (91.6%)	846 (90.3%)		
AG	315 (9.0%)	177 (8.2%)	87 (9.3%)		
GG	14 (0.4%)	5 (0.2%)	4 (0.4%)		
NR3C2 SNP rs2070951				Chi^2^ = 1.5, *p* = 0.46	Chi^2^ = 0.43, *p* = 0.81
CC	816 (23.4%)	548 (25.4%)	229 (24.4%)		
CG	1,729 (49.6%)	1,084 (50.3%)	450 (48.0%)		
GG	939 (27.0%)	523 (24.3%)	258 (27.5%)		
NR3C1 transcript	–	–	6.9 (0.2), 6.0–7.6	–	–
NR3C2 transcript	–	–	7.1 (0.3), 6.1–8.0	–	–
Cortisol (nmol/ml)*	335.5 (131.3), 16–970.4	307.6 (119.1), 14.2–993.6	346.6 (138.3), 52.7–938.5	*T* = 8.2, *p* < 0.001	*T* = − 2.4, *p* = 0.02
BMI***	28.2 (5.2), 15.6–54.4	–	27.4 (4.6), 17.7–53.6	–	*T* = 3.9, *p* < 0.001
BP***	1524 (44.1%)	–	374 (39.2%)	–	Chi^2^ = 7.1, *p* = 0.008
BP medication***	1159 (33.6%)	–	270 (28.3%)	–	Chi^2^ = 9.0, *p* = 0.003
Type 2 diabetes***	344 (10%)	–	16 (1.7%)	–	Chi^2^ = 67.2, *p* < 0.001
Mean hippocampal volume in mm^3^ (*n* = 822)	–	–	7950 (779), 5635–10,194	–	–

*t* test was used for metric data and Chi^2^-test for categorical data

Verbal memory score and current depressive symptoms could not be compared between cohorts as different measurements were used in TREND (verbal memory: *NAI* current depressive symptoms: PHQ-9) and LEGENDE (verbal memory: VLMT, current depressive symptoms: BDI–II); *BMI* body mass index, *MDD*: major depressive disorder; *CTQ* Childhood Trauma Questionnaire, *BP* blood pressure

*Cortisol measured in START-2, not LEGENDE

**For the main analysis between SNP and verbal memory; sample sizes for cortisol, hippocampal volume and gene transcripts are different

***Not measured in LEGENDE

In both cohorts, the SNPs rs56149945 and rs2070951 were in Hardy–Weinberg equilibrium (all *p* > 0.07).

### Direct associations between SNP rs56149945, cortisol and verbal memory in START-2/LEGENDE and TREND

**Fig. 1:**
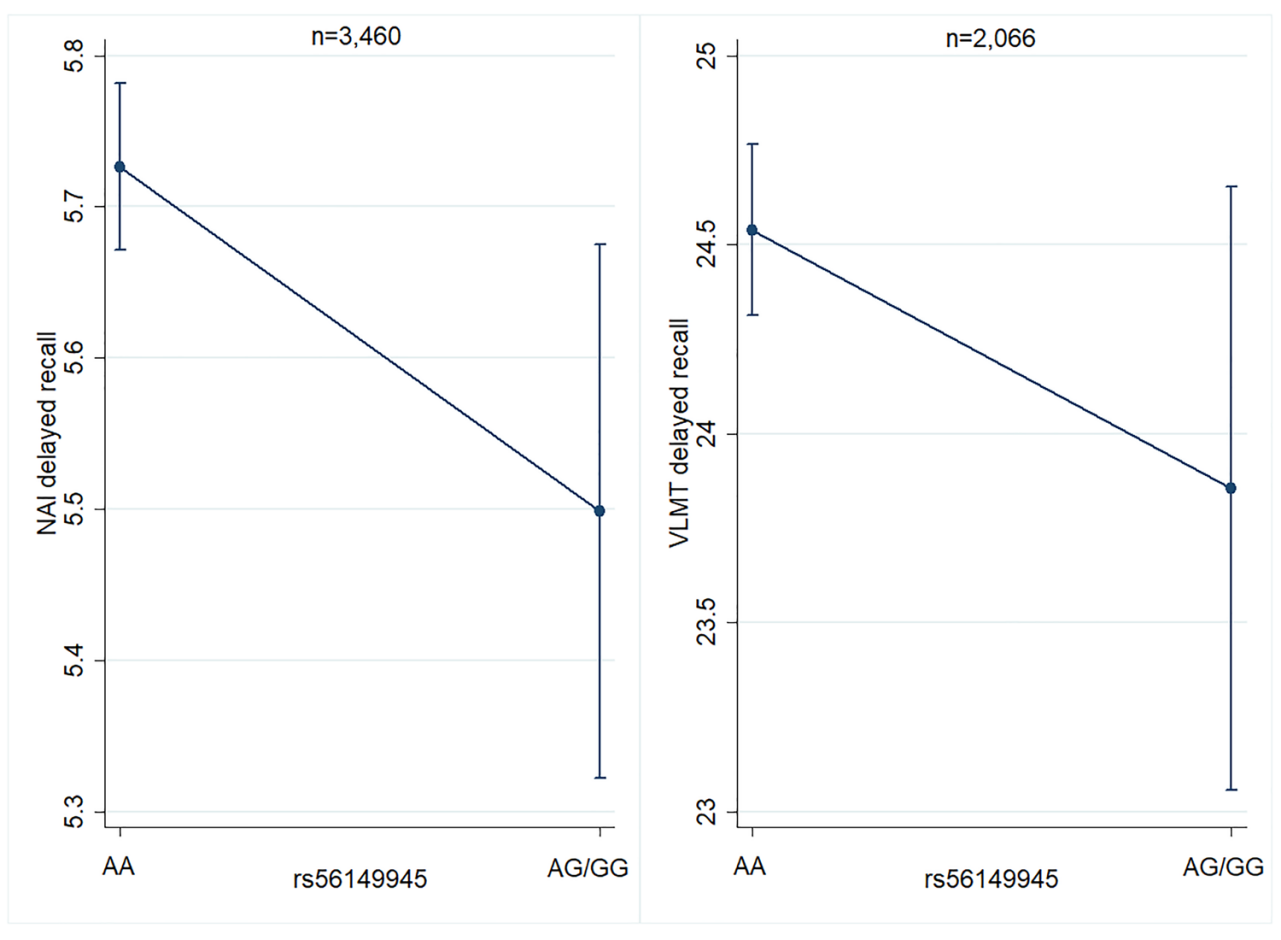
Direct effect of *NR3C1* SNP rs56149945 on delayed verbal memory in TREND and LEGENDE adjusted for age, sex, educational level, current depression score, genetic PCs and genetic batch. Adjusted means with 95% confidence intervals. The G-allele of rs56149945 was associated with lower delayed recall score for NAI in TREND as well as for VLMT in LEGENDE

**Fig. 2:**
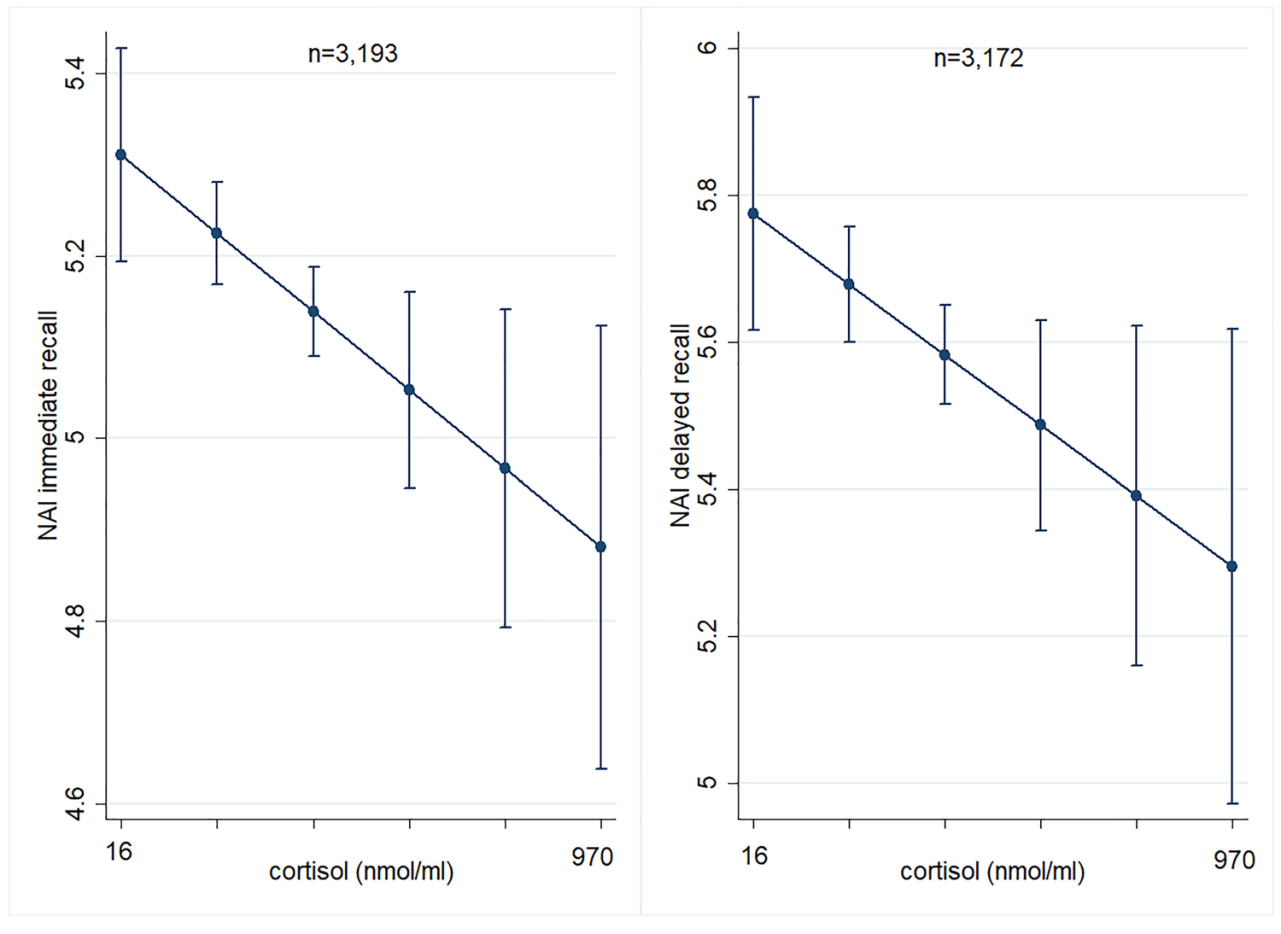
Direct effect of cortisol levels on verbal memory (immediate and delayed recall) in TREND corrected for age, sex, smoking status, alcohol consumption, educational level, current depression score, fasting time, time of blood drawwaist-to-height ratio, HBA1C, white blood cells, red blood cells, and platelets. Adjusted means with 95% confidence intervals. Higher cortisol levels were significantly associated with lower verbal memory score for immediate as well as for delayed recall

In TREND, the G-allele of rs56149945 was significantly associated with reduced scores for immediate as well as delayed recall (immediate: β = − 0.14, *p* = 0.028; delayed: β = − 0.23, *p* = 0.017). This could be replicated in LEGENDE using the VLMT, at least for delayed recall (immediate: β = − 0.68, *p* = 0.091; delayed: β = − 0.46, *p* = 0.047; Fig. 1). The G-allel of rs56149945 was associated with higher cortisol levels in the combined sample with the same effect direction in TREND and START-2 (TREND β = 10.4, *p* = 0.092; START-2 β = 10.6, *p* = 0.25; combined sample β = 10.8, *p* = 0.041). Cortisol itself was associated with lower verbal memory score for immediate (β = − 0.0004, *p* = 0.017) as well as for delayed recall (β = − 0.001, *p* = 0.049) in TREND (Fig. 2, Table 2; analysis not available for SHIP-START).

**Table 2:** Direct effects of *NR3C1*/*NR3C2* SNPs, mRNA transcript level and cortisol level on verbal memory in LEGENDE and TREND

Direct effects on verbal memory in LEGENDE and TREND				
	LEGENDE		TREND*	
	Immediate recall (β, CI, *p* value)	Delayed recall (β, CI, *p* value)	Immediate recall (β, CI, *p* value)	Delayed recall (β, CI, *p* value)
rs56149945 (AG/GG)	− 0.68 [− 1.51, 0.14], *p* = 0.091	*− 0.46 [− 0.93, − 0.02], p = 0.047*	*− 0.14 [− 0.26, − 0.01], p = 0.028*	*− 0.23 [− 0.42, − 0.04], p = 0.017*
rs2070951 (G)	0.01 [− 0.30, 0.33], *p* = 0.94	− 0.002 [− 0.17, 0.17], *p* = 0.98	0.02 [− 0.03, 0.08], *p* = 0.45	0.02 [− 0.06, 0.09], *p* = 0.69
NR3C1 transcript*	–	–	− 0.17 [− 0.53, 0.20], *p* = 0.37	*0.53 [0.04, 1.02], p* = *0.024*
NR3C2 transcript*	–	–	0.10 [− 0.24, 0.43], *p* = 0.56	*0.57 [0.10, 1.03], p* = *0.016*
Cortisol**	–	–	*− 0.0004 [− 0.0008, − 0.00008], p = 0.017*	*− 0.001 [− 0.001, − 2.5E−7], p = 0.049*

Interaction results in TREND subsample			
Interaction	NAI immediate recall (β, *p* value)	NAI delayed recall (β, *p* value)	Hippocampal volume (β, *p* value)
rs56149945 × NR3C1 mRNA	0.84, *p* = 0.18	*2.16,* *p* *= 0.013*	−* 594,* *p* = *0.023*
rs56149945 × cortisol level	*− 0.003,* *p* *= 0.005*	− 0.002, *p* = 0.22	*− 1.66,* *p* *= 0.012*
NR3C1 mRNA × cortisol level	0.002, *p* = 0.07	0.0003, *p* = 0.83	*− 1.29,* *p* *= 0.012*

Interaction effects between SNP rs56149945, *NR3C1* mRNA transcript level and cortisol level on verbal memory and hippocampal volume in the TREND subsample; significant results are highlighted in italic (*p* < 0.05 in discovery sample TREND, *p* < 0.1 in replication sample LEGENDE)

*Gene expression only available in TREND subsample

**Cortisol not available in LEGENDE; β estimates, confidence intervals (CI) and *p* values; (AG/GG) is the effect genotype group of rs56149945; (G) is the effect allele of rs2070951; linear regression analyses were performed for recall score as outcome

### Biological layers analyses for NR3C1 on hippocampal volume and verbal memory in the TREND subsample

The SNP rs56149945 was not associated with the mRNA transcript level of *NR3C1* and thus did not act as expression quantitative trait loci in our sample. However, higher expression levels of the *NR3C1* mRNA transcript were significantly associated with higher delayed recall scores (β = 0.53, *p* = 0.024). Likewise, higher expression of the *NR3C1* mRNA transcript was associated with larger hippocampal volume on a suggestive significance level (β = 172, *p* = 0.075), although the SNP rs56149945 was not directly associated with hippocampal volume in this subsample. There was no association between cortisol levels and hippocampal volume. Analyzing SNP, gene expression and cortisol in a joint model, only higher expression levels for *NR3C1* remained significant for delayed recall (β = 0.50, *p* = 0.062) as well as for hippocampal volume (β = 229, *p* = 0.033). There was further no significant association between verbal memory score and hippocampal volume.

**Fig. 3:**
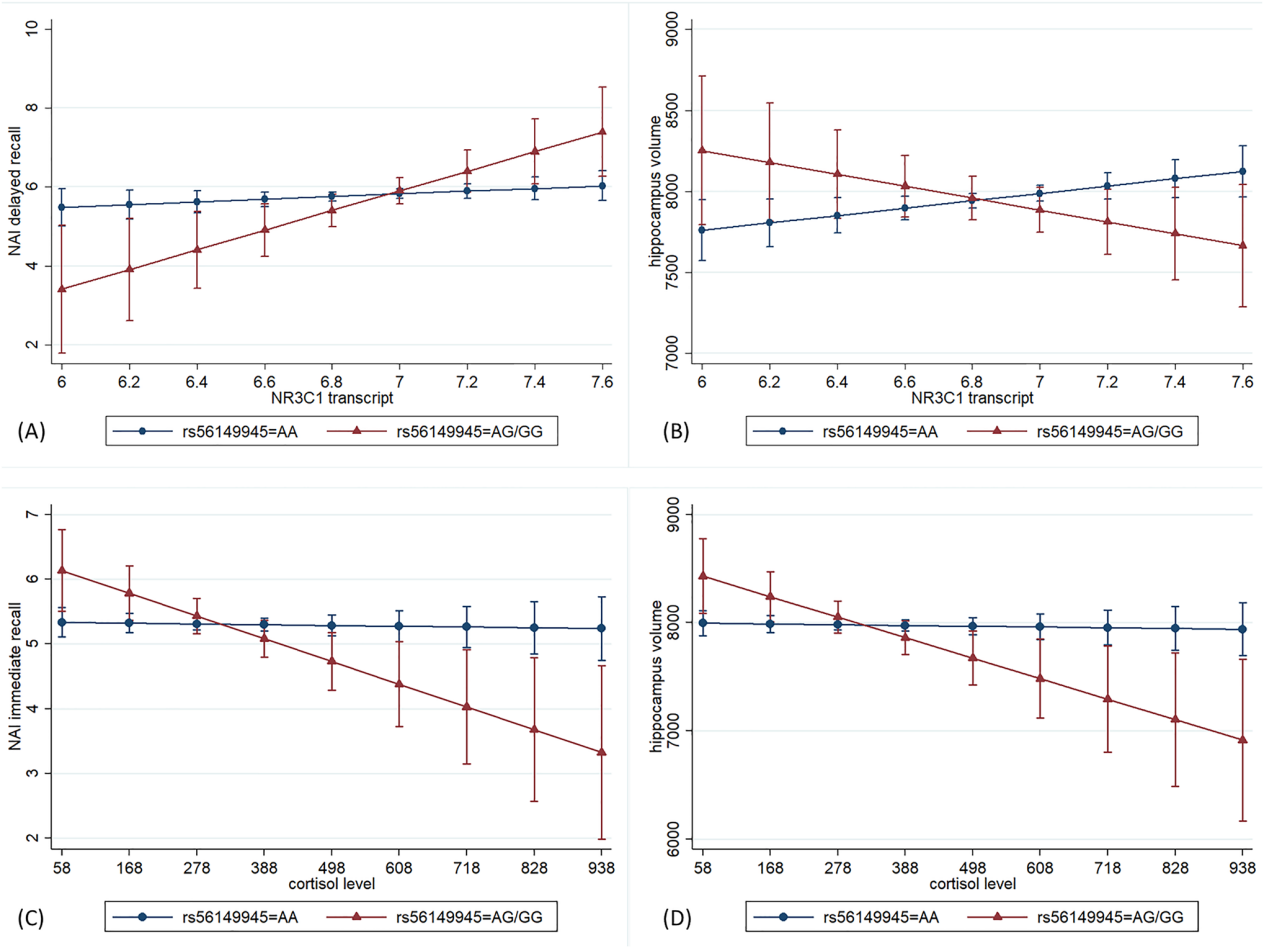
Significant interaction effects on verbal memory and hippocampal volume in the TREND subsample. Adjusted means with 95% confidence intervals. **A** Interaction between NR3C1 SNP rs56149945 and NR3C1 mRNA transcript on delayed recall corrected for time of blood sampling, RNA integrity number, RNA amplification batch, white blood cells, red blood cells, platelets, mean platelet volume, monocytes, lymphocytes, basophils, neutrophils, eosinophils, haematocrit, smoking status, BMI, educational level, current depressive symptoms, age, sex, and genetic PCs (*n* = 930). The negative effect of the G-allele of rs56149945 on delayed recall was reversed under the condition of a high expression level of NR3C1 transcript. **B** Interaction between NR3C1 SNP rs56149945 and NR3C1 mRNA transcript on hippocampal volume corrected for intracranial volume, time of blood sampling, RNA integrity number, RNA amplification batch, white blood cells, red blood cells, platelets, mean platelet volume, monocytes, lymphocytes, basophils, neutrophils, eosinophils, haematocrit, smoking status, BMI, educational level, current depressive symptoms, age, sex and genetic PCs (*n* = 784). The G-allele of rs56149945 was beneficial in case of a low NR3C1 mRNA transcript level, the AA-genotype in case of a high transcript level. **C** Interaction between NR3C1 SNP rs56149945 and cortisol level on immediate recall corrected for age, sex, smoking status, alcohol consumption, educational level, current depression score, fasting time, time of blood draw, waist-to-height ratio, HBA1C, white blood cells, red blood cells, platelets and genetic PCs (*n* = 824). The negative effect of the G-allele of rs56149945 was even stronger under a high cortisol level. An effect of the cortisol level for the AA genotype was not observed. **D** Interaction between NR3C1 SNP rs56149945 and cortisol level on hippocampal volume corrected for intracranial volume, age, sex, smoking status, alcohol consumption, educational level, depressive symptoms, fasting time, time of blood draw, waist-to-height ratio, HBA1C, white blood cells, red blood cells, platelets and genetic PCs (*n* = 699). The effects were similar to those on verbal memory [compare (**C**)]

Taking the three biological layers SNP variation, mRNA transcript and cortisol levels, we also wanted to explore their interaction effects on verbal memory and hippocampal volume in the TREND subsample (Table 2). The interaction between rs56149945 and *NR3C1* mRNA transcript turned out to be statistically significant for delayed recall scores (β = 2.16, *p* = 0.013; Fig. 3) as well as for hippocampal volume (β = − 595, *p* = 0.023; Fig. 3). In case of verbal memory the negative effect of the G-allele of rs56149945 could be reversed under the condition of a high *NR3C1* mRNA transcript level. For hippocampal volume, the negative effect of the G-allele could be reversed in case of low *NR3C1* mRNA transcript level in blood. The interaction between rs56149945 and cortisol level was significant for immediate recall score (β = − 0.0031, *p* = 0.005) as well as for hippocampal volume (β = − 1.66, *p* = 0.013) with the G-allele showing even worse effects in case of higher cortisol levels (Fig. 3). Similar results could be observed for the interaction between *NR3C1* mRNA transcript and cortisol levels on verbal memory and the hippocampal volume. This interaction became significant for hippocampal volume (β = − 1.50, *p* = 0.044) and immediate recall score (β = 0.003, *p* = 0.047).

### Validating direct associations for mRNA transcript (NR3C2) and SNP rs2070951 on cortisol, verbal memory and hippocampal volume

For rs2070951, there were no significant direct associations on verbal memory in this cross-sectional setting (Table 2). Similar to the *NR3C1* mRNA transcript, the *NR3C2* mRNA transcript was significantly associated with higher delayed recall score (β = 0.57, *p* = 0.016). The SNP rs2070951 was not associated with the expression level of *NR3C2* mRNA transcript and did not act as an eQTL in our sample. The G-allele of rs2070951 was associated with higher cortisol levels in START-2 but slightly missed significance in the combined sample (TREND β = 2.656, *p* = 0.32; START-2 β = 6.55, *p* = 0.034; combined sample β = 3.77, *p* = 0.052). There was further no association between *NR3C2* mRNA transcript and cortisol levels. The G-allele of rs2070951 was associated with reduced hippocampal volume in the TREND subsample (β = − 52.57, *p* = 0.036) whereas the *NR3C2* mRNA transcript revealed no effect on hippocampal volume. None of the above interactions between the SNP rs2070951, *NR3C2* mRNA transcript and cortisol became significant in TREND.

## Discussion

Complex phenotypes, such as cognition and memory function are regulated by a complex interplay between different genetic and epigenetic features. In this paper, we demonstrated the importance of the *NR3C1* gene on this system in two independent general population samples and highlighted biological interactions between *NR3C1* SNP variation, mRNA transcript level and cortisol not only on verbal memory, but also on hippocampal volume. Similar associations with *NR3C2* SNP and mRNA transcript revealed a different pattern of associations indicating different mechanisms of the GR and MR system.

In detail, SNP variation of rs56149945 was associated with verbal memory in both samples with the G-allele being associated with lower delayed memory performance. Although rs56149945 could be associated to various psychiatric disorders in the past [23, 37, 38] as well as cognitive features related to preterm birth [39], we are the first to report an effect on verbal memory of neutral words in the general population. The SNP itself is located in the first exon of the *NR3C1* gene as a missense variant that causes an amino acid change from asparagine to serine or isoleucine (https://www.ncbi.nlm.nih.gov/, Fig. S2). The SNP also showed a significant effect on cortisol in the combined sample with the G-allele associated with higher cortisol levels. This links genetic variation in the GR gene to the level of glucocorticoids, which may be caused by either differences in binding affinities based on the genetic variation or a feedback regulation of cortisol secretion of *NR3C1*. Our data also confirm the established role of cortisol in memory function, especially in response to stress [19]. Participants with higher cortisol levels revealed a lower verbal memory score also for the recall of neutral words without an external stressor. The associations also remained stable when adjusting for the chronic psychological stressors depression and childhood traumatization.

Our second investigated SNP rs2070951 of the *NR3C2* gene, revealed no significant effect on memory in this cross-sectional setting. Nevertheless, we were able to demonstrate an effect of the C-allele on memory decline in a longitudinal setting in a previous paper which shows that longitudinal and cross-sectional biological mechanism ot always act in the same way [14]. In line with this, Langer et al. [15] demonstrated an interaction effect between the SNP and cortisol levels on learning strategies. Also, *NR3C2* haplotypes including this SNP showed differences in cortisol-induced gene expression in a sample of 166 teachers [40]. However, investigating the single SNP effect we could not replicate an association with *NR3C2* gene expression although the SNP precedes the start codon of the *NR3C2* gene and lies in the 5 ‘ untranslated region which harbors the binding sites for transcription enzymes (https://www.ncbi.nlm.nih.gov/, Figure S3). Results for the association between rs2070951 and cortisol level are by now contradicting. Langer and colleagues [15] reported a significant association with higher cortisol levels for the CC-allele carriers in a sample of *n* = 74 healthy males, whereas Muhtz and his group [41] found a significant effect for the G-allele and higher saliva cortisol in *n* = 133 healthy subjects which is in line with our results.

Even though, we found no evidence for rs56149945 to be an eQTL, a study by Jewell and colleagues [42] could link this polymorphism to altered gene regulation which might modulate the effect on verbal memory. The mRNA transcript of *NR3C1* revealed a significant effect on delayed verbal memory recall in the TREND subsample with higher transcript levels associated with higher recall scores. This was also the case for the *NR3C2* mRNA transcript, although rs2070951 was no eQTL in our sample. Previous research in mice and rats supported the association between transcript levels of *NR3C1* and *NR3C2* and memory. Overexpression of *NR3C2* was found to enhance memory consolidation in mice [43, 44]. Ferguson and Sapolsky found that overexpression of *NR3C2* as well as transdominat *NR3C1* blocks the impairing effect of glucocorticoids on memory in rats [45]. For *NR3C1,* lower expression was associated with reduced picture recognition in male adults [8]. Thus, these findings were suggestive of independent effects of the SNPs and the corresponding mRNA transcript on memory.

In contrast to the *NR3C2* mRNA transcript, the *NR3C1* mRNA transcript level was additionally positively associated with hippocampal volume. Regarding the two SNPs, only the G-allele of rs2070951 was associated with reduced hippocampal volume. Only Gerritsen and colleagues [46] identified a SNP of the *NR3C2* gene as associated with hippocampal volume but only in interaction with childhood trauma. Thus, for both findings we are the first to report a respective association. In combined analysis including rs56149945, NR3C1 transcript and cortisol, only the transcript level remained significantly related to memory and hippocampal volume making it a key regulator in this system.

Additionally, we could demostrate that the different biological layers related to *NR3C1* do not act independently. We found interactions between SNP rs56149945, gene expression for *NR3C1,* and cortisol levels on verbal memory as well as hippocampal volume, but not for *NR3C2*. For *NR3C1* there were associations between SNP*transcript, SNP*cortisol and transcript*cortisol for verbal memory as well as for hippocampal volume. For the interaction between cortisol level and SNP the negative effect of the G-allele of rs56149945 could be reinforced under the condition of high cortisol levels. This might be a hint for an interaction between *NR3C1* genetics and stress response on memory also found for several other *NR3C1* and *NR3C2* SNPs [47]. The results for the SNP*transcript interaction were different for the endpoints memory and hippocampal volume. Under the condition of a high expression of *NR3C1* the negative effect of the G-allele of rs56149945 on delayed verbal memory could be reversed whereas for hippocampal volume high levels for *NR3C1* were only beneficial for the AA-genotype. Regarding the last interaction, a high cortisol level in combination with an also higher *NR3C1* mRNA transcript level showed a positive effect on immediate recall whereas for hippocampal volume low cortisol levels and higher *NR3C1* transcript levels were beneficial. Findings like this emphazise the complexity of this system modulated by the combination of genetic and epigenetic factors.

There are different hypotheses to explain the interaction effects found for *NR3C1*. According to our data, different components of *NR3C1* mechanism closely interact to affect memory and hippocampal volume. Interestingly, those interactions could not be observed for *NR3C2* confirming the hypothesis of *NR3C1* being involved in cortisol response [48] and inducing anti-inflammatory effects. This shows how the system is able to counteract under adverse conditions. Counterintuitive results from interaction analyses for *NR3C1* might be explained by a u-shaped association between stress and memory function. Both, reduced and increased cortisol levels, might impair memory [49] function which forces the *NR3C1* system to act sensitively under minimal changes of the environment.

Interactions between *NR3C1* genetic variants and trauma on memory and hippocampl volume have been identified in the past, but only on the level of genetic profile scores including the combination of multiple SNPs. Mahli et al. [20] found a gene-environment interaction between trauma and a genetic variant of *NR3C1* on hippocampal volume in adolescent girls. Likewise, Pagliaccio [21] identified the same interaction using a genetic profile score, including variants from *CRHR1*, *NR3C2*, *NR3C1*, and *FKBP5*, instead of a single SNP in a sample of 120 preschool aged children. Hartling and colleagues [50] used a slightly different genetic profile score for HPA-axis genes, including *NR3C1*, *CRHR1* and *FKBP5*, to predict facial emotion recognition in the interaction with childhood trauma.

Although our data draw a complex picture of the effects of different biological factors on memory at least for the effect of *NR3C1* SNP and transcript (Figure S6), our conclusions were limited by various factors. We were not able to replicate all findings presented, especially those for the gene expression levels, as these data were only available in a subsample of TREND. Even though, we tried to account for biological differences regarding the TREND subsample, such as age, sex, educational level, smoking status or blood levels, there were significant differences between the full TREND sample and the subsample regarding important biological factors. These differences were influenced by the fact that the subsample contained mainly non-diabetic subjects. As a result, subjects of the TREND subsample showed a lower BMI, as well as lower rates of hypertension and hypertensive medication as can be seen in Table 1. In further studies it might be interesting to additionally investigate the impact of these factors. Additionally, there might be unmeasured factors one might have accounted for, such as post-traumatic stress-disorder, vitamin D or chronic diseases that are known to have an impact on memory. Furthermore, our main outcome verbal memory was measured using different methods, which limits comparability. Yet, this strengthenes the validity of our findings because the effect was replicated using different methologies for cognitive performance. In addition, our findings for cortisol levels are based on a single occasion measurement with varying times of blood samples, which is vulnerable to influences of circadian rhythm. Also, different epigenetic layers are not present in our analysis, such as gene-methylation or small regulatory RNAs which are also known to modulate HPA axis function [51]. Finally, we were not able to support our hypotheses with further laboratory experiments, such as psychological stressors or a dexamethasone suppression test, as our data come from two cross-sectional general population samples. Nevertheless, it is up to various other groups now to validate our findings in independent data and/or experimental settigs to complete this picture of the effects of GR variation on memory function. Furthermore, it would be interesting to include markers of inflammation into the model as both, the GR and the MR, mediate inflammatory as well as anti-inflammatory effects [52].

In sum, we provided a mechanistic concept of the interaction between various biological layers spanning GR function and its effects on memory.

## Supplementary Information

Below is the link to the electronic supplementary material.

Supplementary file1 (PDF 1085 KB)
