# Supplementary material for: The interplay between genetic variation and gene expression of the glucocorticoid receptor gene NR3C1 and blood cortisol levels on verbal memory and hippocampal volumes
Source: Eur Arch Psychiatry Clin Neurosci. 2022 May 17;272(8):1505–16. doi: 10.1007/s00406-022-01420-w (PMC9653325; doi:10.1007/s00406-022-01420-w)
Supplement: Supplementary file 1 — Supplementary file1 (PDF 1085 KB) [file 406_2022_1420_MOESM1_ESM.pdf]

SUPPLEMENTARY MATERIAL FOR: The interplay between genetic variation and gene expression of the glucocorticoid receptor gene NR3C1 and blood cortisol levels on verbal memory and hippocampal volume

Sandra Van der Auwera, Johanna Klinger-König, Katharina Wittfeld, Jan Terock, Anke Hannemann, Robin Bülow, Matthias Nauck, Uwe Völker, Henry Völzke, Hans Grabe

SUPPLEMENTARY METHODS

Blood measurements:

Blood samples in START-2 and TREND were taken from the cubital vein. Serum and plasma samples were stored at -80°C in the Integrated Research Biobank of the University Medicine Greifswald and used in accordance with its regulations.

Glycated hemoglobin (HbA1c) concentrations were quantified by high-performance liquid chromatography (Bio-Rad Diamat, Munich, Germany). White blood cell count (WBC), red blood cell count (RBC) and platelet count (PLT) were measured on the XT2000, XE 5000 or SE9000 analyzers from Sysmex (Sysmex Deutschland GmbH, Norderstedt, Germany) or on the Advia 2120i (Siemens Healthcare Diagnostics, Eschborn, Germany).

Serum cortisol was measured using a chemiluminescence-immunoassay on the AVIDA CENTAUR XP (Siemens Healthcare Diagnostics, Eschborn, Germany). The coefficients of variation for the cortisol measurements were 5.74% and 6.94% for low and high dose control material in START-2 and 9.11% and 7.47% for low and high dose control material in TREND, respectively. Pregnant woman and subjects taking hormones or synthetic glucocorticoids (ATC G03 and H02) were excluded from the cortisol analyses.

Gene expression:

Briefly, fasting whole-blood samples were collected and stored in PAXgene Blood RNA Tubes (BD). RNA was prepared using the PAXgeneTM Blood miRNA Kit (QIAGEN, Hilden, Germany). Purity and concentration of RNA were determined using a NanoDrop ND-1000 UV-Vis Spectrophotometer (Thermo Scientific). For quality control of RNA samples, all preparations were analysed using a 2100

Bioanalyzer and RNA 6000 Nano Lab Chips (both from Agilent Technologies, Santa Clara, CA, USA) according to the manufacturer's instructions. Samples exhibiting an RNA integrity number (RIN) less than seven were excluded from further analysis. Whole–blood transcriptome profiling of the samples was performed using the Illumina HumanHT-12 v3 BeadChip array.

MRI data in TREND

For structural magnetic resonance imaging (MRI) data of the head, participants were scanned with a 1.5 Tesla MRI scanner (MAGNETOM Avanto; Siemens Healthcare, Erlangen, Germany) with a T1 weighted magnetization prepared rapid acquisition gradient-echo (MPRAGE) sequence and the following parameters: axial plane, repetition time=1900 msec, echo time=3.4 msec, flip angle=15° and original resolution of 1.0x1.0x1.0mm³, matrix 256x176, bandwidth 130Hz/Pixel. Images were processed using the segmentation software Freesurfer (33) (v7.1.1) to estimate the volume of the hippocampus of the lelft and right hemisphere (34).

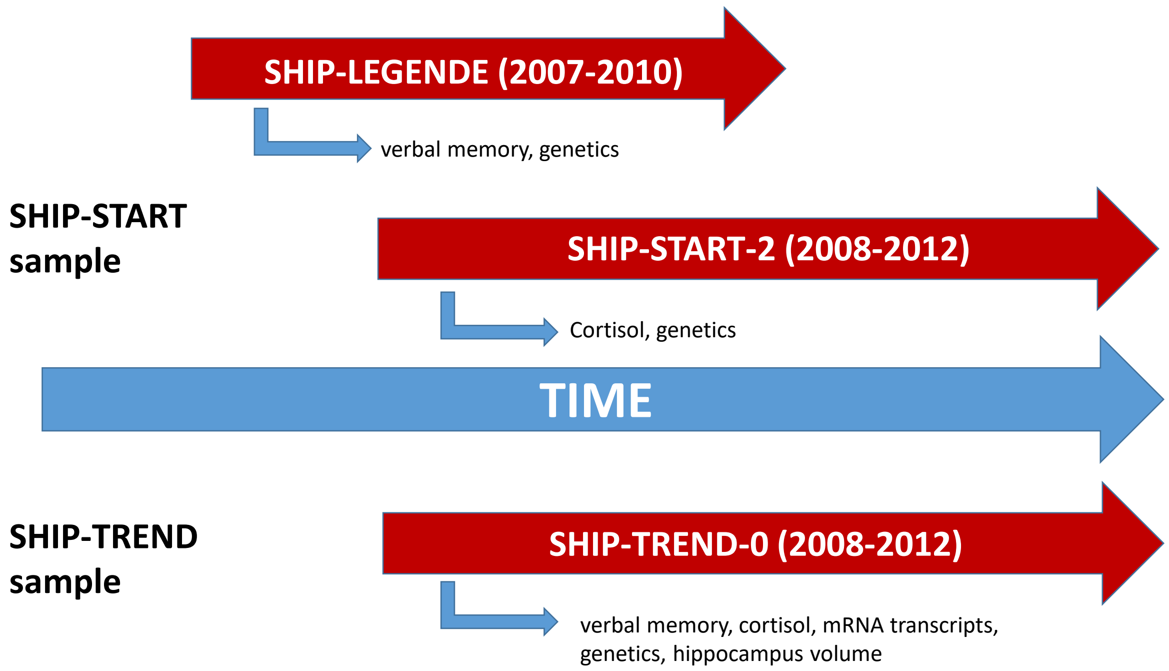

Figure S1. Graphical overview of the investigated samples and the variables available in each sample (SHIP-START-1 with n=3,300 from 2002 to 2006; SHIP-START-2 with n=2,333 from 2008 to 2012 and SHIP-START-3 with n=1,718 from 2014 to 2016;).

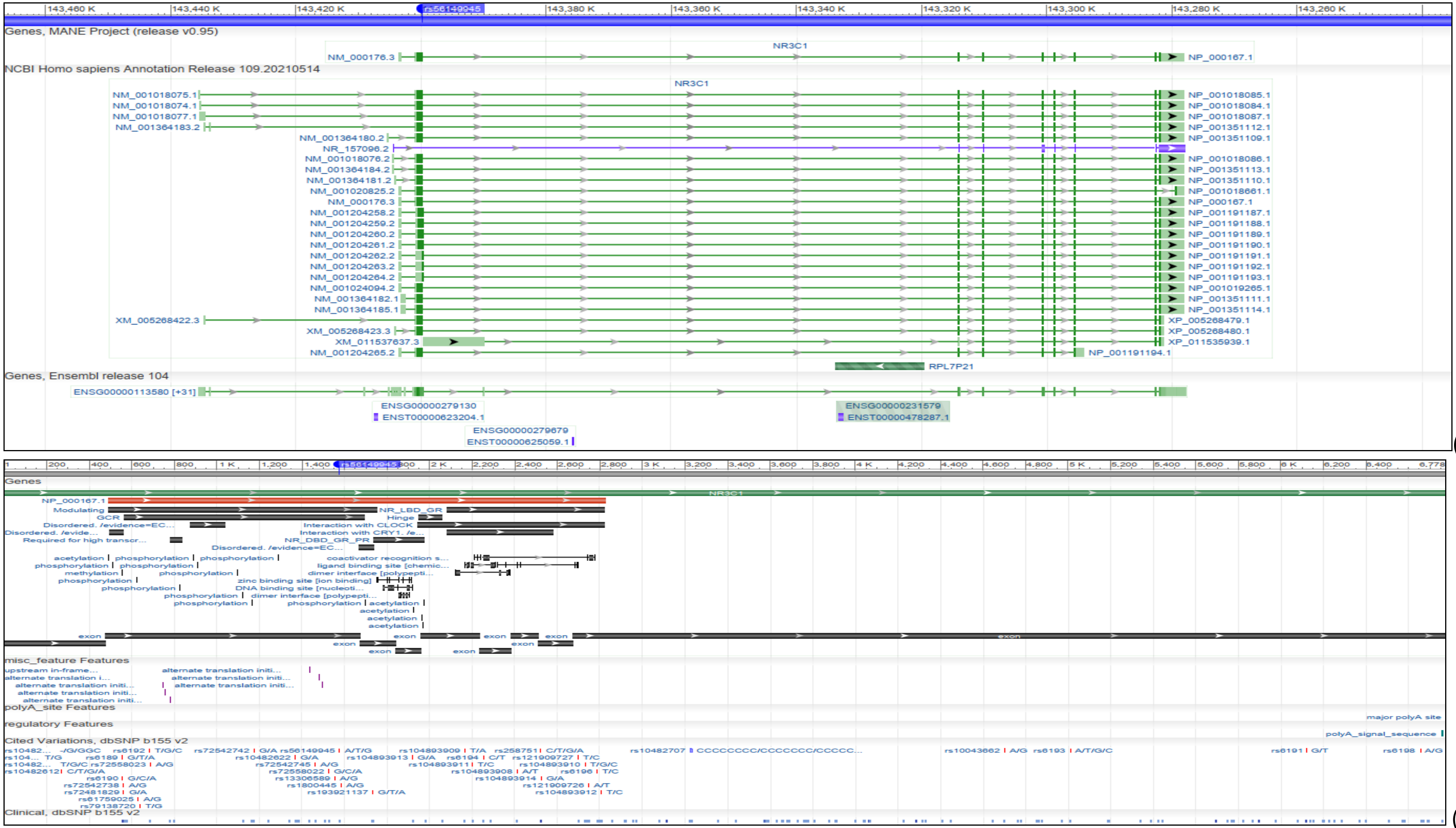

(A)

(B)

Figure S2. *NR3C1* plots from NCBI (https://www.ncbi.nlm.nih.gov/) (A) plot of the *NR3C1* gene on chromosome 5. (B) Location of the transcript and SNP rs56149945 within the gene.

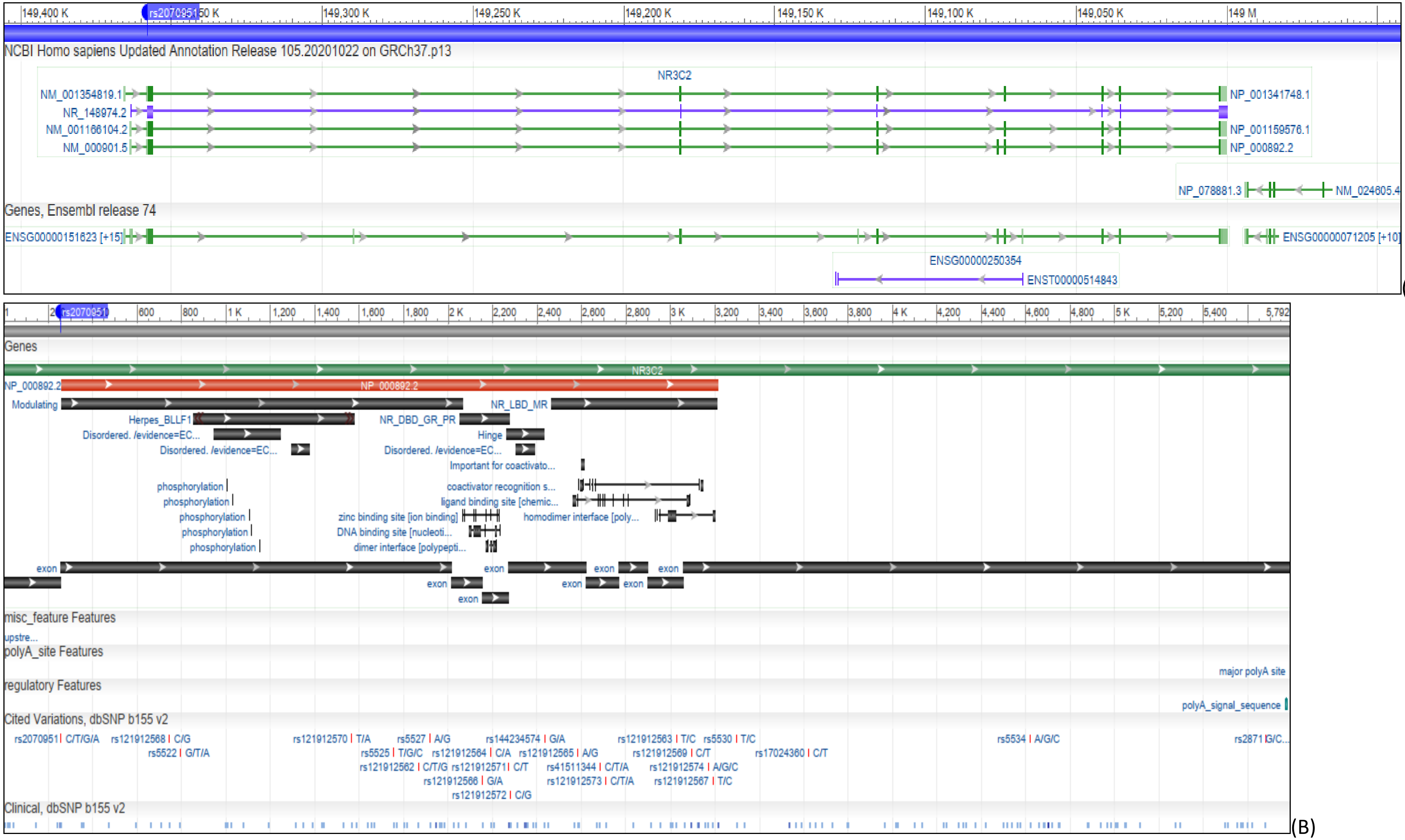

(B)

Figure S3. *NR3C2* plots from NCBI (https://www.ncbi.nlm.nih.gov/) (A) plot of the *NR3C2* gene on chromosome 4. (B) Location of the transcript and SNP rs2070951 within the gene.

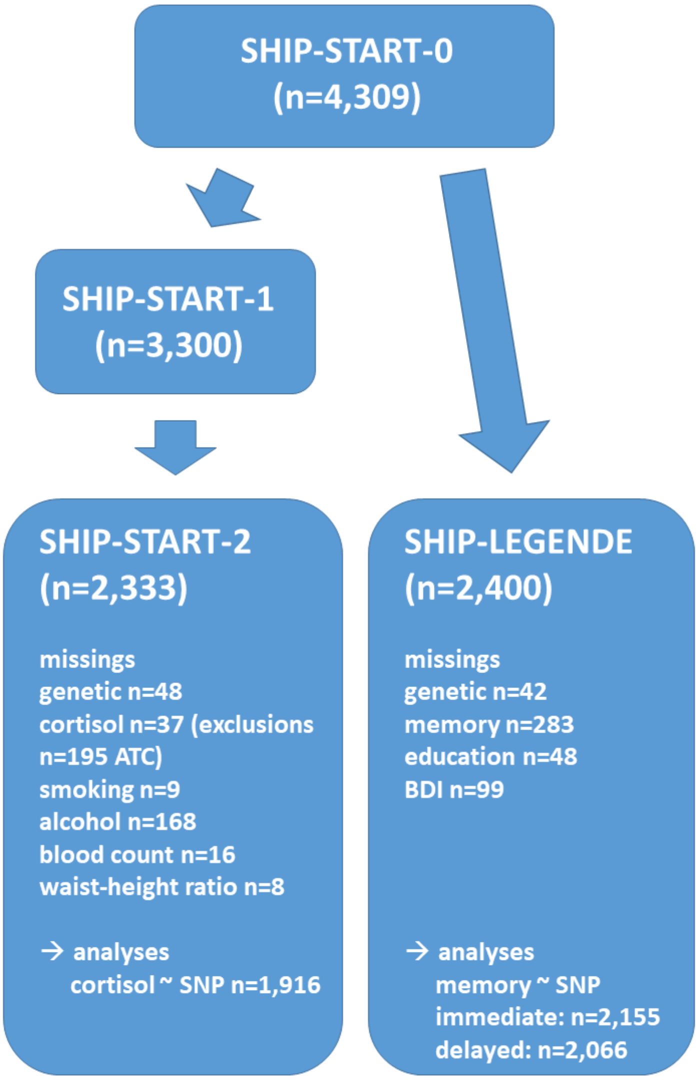

Figure S4. Drop-outs and final sample size for the SHIP-START sample.

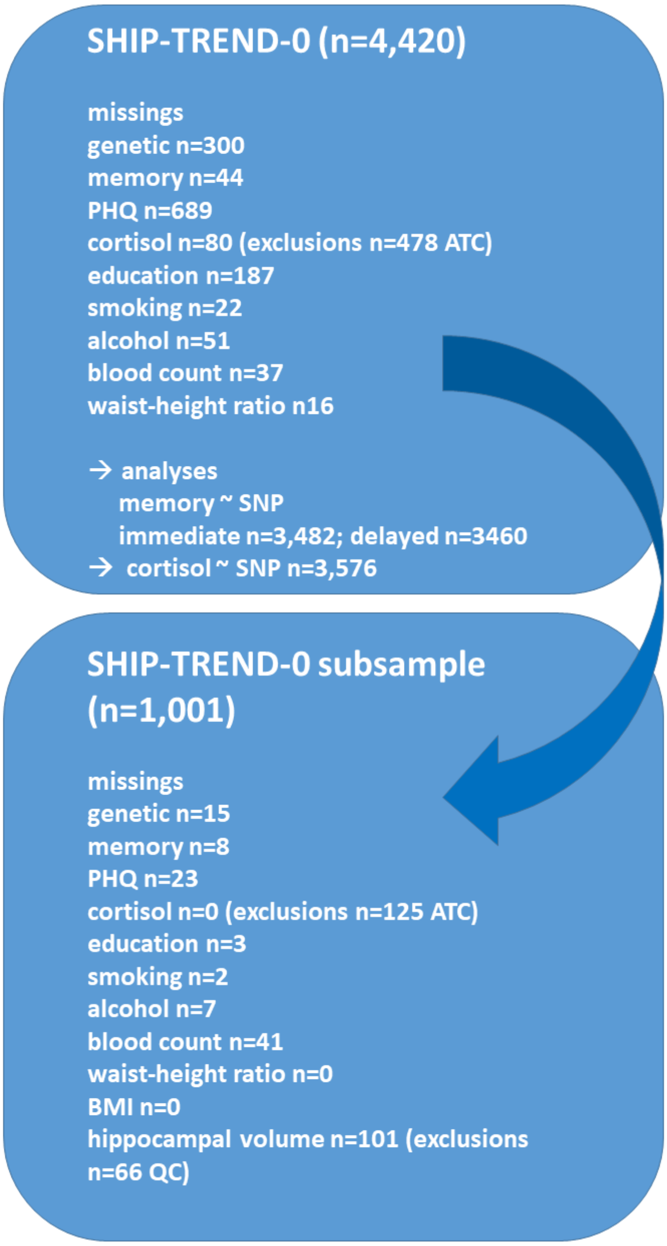

Figure S5. Drop-outs and final sample size for the SHIP-TREND sample.

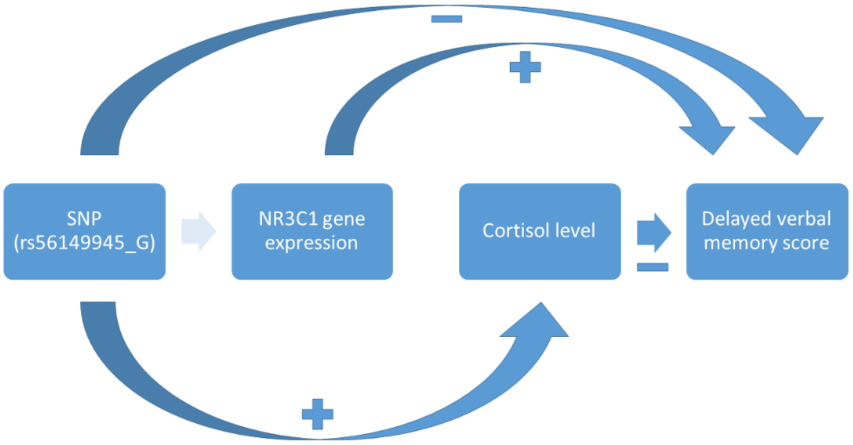

Figure S6. Consistent picture of the effects of different biological factors on memory for the effect of *NR3C1* SNP and transcript.

The G-allele of rs56149945 exhibited a negative effect on delayed verbal memory score and a positive effect on higher cortisol levels which itself was associated with lower verbal memory score. Higher gene expression levels of NR3C1 were associated with higher verbal memory score.
